# An Observational Study of the First 100 Patients Undergoing Nocturnal Every-Other-Day Online Hemodiafiltration: Clinical Outcomes and Patient and Technique Survival

**DOI:** 10.3390/jcm14010251

**Published:** 2025-01-03

**Authors:** Francisco Maduell, Víctor Joaquín Escudero-Saiz, Lida Maria Rodas, Elena Cuadrado, Laura Morantes, Marta Arias-Guillen, Néstor Fontseré, Nayra Rico, José Jesús Broseta

**Affiliations:** 1 Nephrology and Renal Transplantation, Hospital Clínic de Barcelona, 08036 Barcelona, Spain; vjescudero@clinic.cat (V.J.E.-S.); lmrodas@clinic.cat (L.M.R.); ecuadrado@clinic.cat (E.C.); morantes@clinic.cat (L.M.); marias@clinic.cat (M.A.-G.); fontsere@clinic.cat (N.F.); jjbroseta@clinic.cat (J.J.B.); 2 Biochemistry and Molecular Genetics Department—CDB, Hospital Clínic de Barcelona, 08036 Barcelona, Spain; nrico@clinic.cat

**Keywords:** dialysis adequacy, every other day, nocturnal dialysis, online hemodiafiltration, survival

## Abstract

**Background**: High-volume online hemodiafiltration (OL-HDF) has proven to be the most efficient dialysis modality and to offer better clinical outcomes in patients on hemodialysis. Longer and more frequent dialysis sessions have demonstrated clinical and survival benefits. **Methods**: A single-center observational study of the first one hundred patients on nocturnal every-other-day OL-HDF was conducted with the aim of reporting the experience with this treatment schedule and evaluating analytical and clinical outcomes as well as the patient and technique survival. **Results**: Nocturnal OL-HDF on alternate days was highly accepted, with no adverse symptoms, good clinical tolerance, and maintained active work in 62%. Kt, and the convective volume increased from 67.6 ± 12 L to 105.4 ± 11.7 L, and from 27.1 ± 4.6 L to 48.1 ± 6.4 L, respectively, from the baseline to 24 months. An improved calcium–phosphate balance and blood pressure control were observed, as the use of phosphate binders and antihypertensive medications decreased from 76.7% to 3.3% and from 56.7% to 28.3%, respectively. Furthermore, 58.3% of patients required phosphate supplementation in the dialysis fluid to prevent intradialytic hypophosphatemia. Additionally, doses of iron and erythropoiesis-stimulating agents were reduced. The global patient survival was 94% at the end of the follow-up. It was higher in those on the transplant waiting list, with 98.1% survival compared to 84.6% in non-wait-listed patients at 24 months. The main reason for treatment discontinuation was kidney transplantation, accounting for 78.4% of the 88 withdrawals, while death was the leading cause of discontinuation in non-listed patients (41.6%). **Conclusions**: Nocturnal every-other-day OL-HDF is a well-tolerated dialysis regimen that offers significant clinical benefits, which may positively impact morbidity and mortality. Additionally, it allows patients to integrate the treatment into their social and occupational lives.

## 1. Introduction

Patients with dialysis-dependent chronic kidney disease face higher mortality and morbidity rates, with a life expectancy lower than the general population [1]. Despite the advances in hemodialysis membranes, dialysates, biosensors, and pharmacological treatments, mortality rates have remained at similar figures during the last few decades [2], with around half of hemodialysis patients dying due to cardiovascular causes [3]. Consequently, new dialysis approaches such as an increasing frequency and dialysis duration [4] or the development of higher depurative dialysis techniques are needed to enhance surveillance and cardiovascular outcomes in this population.

Currently, thrice-weekly four-hour hemodialysis schemes have been widely accepted for many reasons, but long-term experiences with more frequent schemes have shown promising outcomes [5,6,7,8,9,10]. The advantages of longer hemodialysis (> 540 min of treatment) have been evaluated by several studies, and they include benefits in terms of blood pressure control, bone mineral metabolism, anemia, the acid–base balance and nutritional status, a reduction in left ventricular hypertrophy (LVH), and an improvement in the left ventricular ejection fraction [11,12,13,14].

Online hemodiafiltration (OL-HDF) integrates diffusion and a high convection, providing the highest clearances for all-sized uremic toxins [15]. Post-dilution high-volume OL-HDF is the hemodialysis technique with more favorable outcomes in terms of reducing the overall and cardiovascular mortality in different randomized trials from different populations [16,17].

To integrate extended nocturnal dialysis with more frequent sessions (every other day), along with the modality offering the highest solute and uremic toxin removal (OL-HDF), a 12-month pilot study was initiated in September 2007. The study concluded that long-term, in-center nocturnal dialysis performed every other day with high-volume OL-HDF could be an effective therapeutic approach that may improve clinical outcomes and facilitate social and occupational rehabilitation [18]. After these positive short-term results, the maintenance of this treatment scheme in the long term was decided. This paper gathers the results of the first 100 included patients on nocturnal, every-other-day, online hemodiafiltration and evaluates the analytical and clinical outcomes and patient and technique survival.

## 2. Materials and Methods

This observational study included the first one hundred patients undergoing nocturnal every-other-day OL-HDF with a recruitment period from September 2007 to July 2023. Patients were previously on standard 4–5 h dialysis thrice weekly for 19 ± 21 months. The underlying kidney diseases were chronic glomerulopathies in 27 patients, nephroangioesclerosis in 17, tubule–interstitial nephritis in 14, chronic kidney disease (CKD) of an unknown etiology in 13, diabetic nephropathy in 10, a urological etiology in 9, polycystic kidney diseases in 5, systemic erythematous lupus in 3, multiple myeloma in 1, and atypical uremic–hemolytic syndrome in 1.

All patients were considered for their inclusion if they were in a stable condition while undergoing hemodialysis, with adequate vascular access and promising prospects for improved occupational, psychological, and social rehabilitation, and gave their written informed consent.

This was an in-center hospital experience. Dialysis sessions were conducted in a general room that accommodated up to 12 patients, with nursing staff always present at a nurse-to-patient ratio of 1:5. Patients did not receive any specialized education or training, and they laid on beds without sound barriers, using the same machines that were utilized during the morning and afternoon shifts.

The baseline OL-HDF parameters were 32–35 mmol/L bicarbonate buffer, 1.4–1.9 m^2^ surface high-flux dialyzers, a blood flow (Qb) of 421 ± 50 mL/min (range of 350–500), a dialysate flow (Qd) of 524 ± 161 mL/min (range of 400–800), an infusion flow (Qi) ranging from 80 to 120 mL/min, and a Fresenius 5008 or 6008 dialysis monitor. Reinfusion was always performed in post-dilutional mode. The vascular accesses were an autologous arteriovenous fistula (AVF) in 92 patients, a prosthetic arteriovenous fistula in 3 patients, and a tunneled catheter in 5 patients.

The dialyzers were not reused in any of the patients. We used 15-gauge needles for all procedures, maintaining standard protocol for securing the needles. The only change made was replacing the usual sticking plaster with a special transparent dressing (3M TegadermTM film). Anticoagulation was achieved using a non-fractionated heparin (25%), low-molecular-weight heparins (73%), an oral anticoagulant (1%), or no anticoagulation (1%).

The following dialysis and urea kinetic parameters were recorded on a quarterly basis: the treatment duration (Td), Qb, convective volume, pre- and post-dialysis weight, Kt estimated by ionic dialysance, urea reduction ratio (URR), single-pool second-generation Daugirdas Kt/V (spKt/V), equilibrated Kt/V (eKt/V), standard Kt/V (stdKt/V), time-averaged concentration (TAC), and normalized protein catabolic rate (nPCR).

Quantitative data are presented as the mean ± the standard deviation, while qualitative data are expressed in terms of absolute and relative frequencies. Each patient served as their own control. An ANOVA for repeated measures was conducted to assess differences in the quantitative variables over the follow-up period. A *p*-value of <0.05 was considered statistically significant. Analyses were performed using SPSS software version 23 (SPSS, Chicago, IL, USA).

## 3. Results

**Figure 1:**
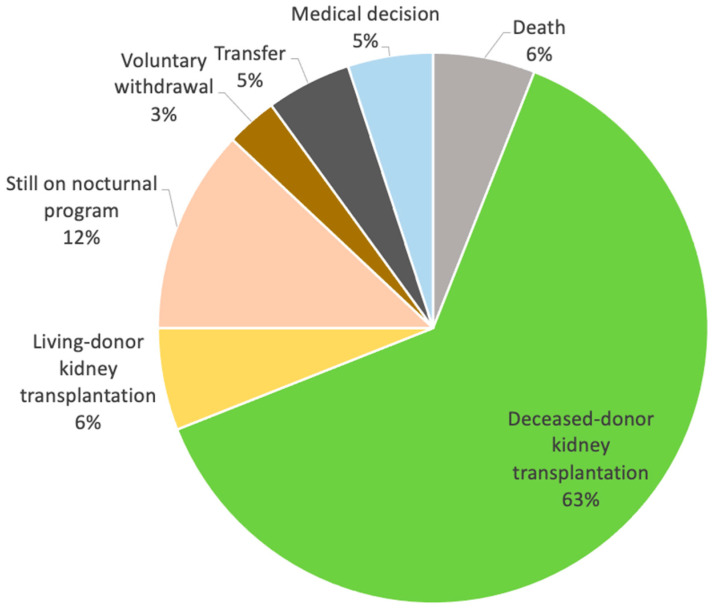
List of withdrawals from the nocturnal, every-other-day OL-HDF program.

A total of 100 patients (31 females), with a mean age of 48 ± 13.8 years (range of 18–82), were included. They received nocturnal, every-other-day OL-HDF treatment, with an average follow-up period of 36.1 ± 34 months (ranging from 2 to 186) until September 2024. During the follow-up period, 88 patients discontinued this dialysis scheme for various reasons, with kidney transplantation being the most common (*n* = 69) (Figure 1). The median follow-up duration was 24 months (*n* = 60), and as a result, the analytical data are reported at the 24-month mark.

At baseline, 62 patients were employed and continued working throughout the nocturnal treatment. The types of jobs held by these patients included administrative positions (seven), school teaching or monitoring (six), legal professions (five), engineering (four), student roles (four), restaurant work (four), hairdressing (three), work with the National Organization of Spanish Blind People (three), technical architects (three), self-employment (three), taxi driving (two), cleaning services (two), religious professions (two), banking (one), biology (one), caregiving (one), carpentry (one), nursing (one), international business (one), museum directorship (one), music (one), pharmacy (one), medicine (one), restaurant supply (one), security guarding (one), and a watchman (one), as well as a writer (one).

### 3.1. Transplantation and Waiting List

Eighty-four patients were on the kidney transplantation waiting list, while sixteen were not. Their mean time on nocturnal hemodialysis was 31.62 ± 27.9 for those on the waiting list and 60.13 ± 52.3 for those who were not.

Out of the 84 patients on the waiting list, 69 (82%) received kidney transplants. Of the remaining fifteen, nine were still in the nocturnal program at the end of the follow-up, three withdrew voluntarily, one died, and two transferred, one to other unit due to a change of address and the other due to organizational reasons. Meanwhile, among the sixteen patients not on the kidney transplantation waiting list, five patients died, four were still in the nocturnal program, three moved to another city, three transferred due to medical decisions, and one withdrew voluntarily.

### 3.2. Dialysis Parameters and Urea Kinetics

The dialysis duration increased from 279.1 ± 28.1 min (range of 180–330 min) at baseline to 478 ± 10.9 min (range of 420–480) with the change in the treatment scheme (Table 1). The mean baseline Qb was 422 ± 34 mL/min and remained stable during the follow-up (415 ± 34 mL/min at 24 months), and the mean baseline Qd was 536 ± 171 mL/min, which decreased to 359 ± 104 mL/min at 24 months. The convective volumes increased from 27.1 ± 4.6 at baseline to 48.1 ± 6.4 at 24 months (Table 1).

All the evaluated urea kinetic parameters showed a significant improvement in the dialysis dose, as illustrated in Table 2. The Kt increased from 67.7 ± 12.0 L to 105.4 ± 11.7 L at 24 months, accompanied by a decrease in the TAC and an increase in the PRU, stdKtV, spKtV, and eKtV (Table 2).

### 3.3. Nutritional Parameters

Patients reported an improved appetite and a tendency to increase their protein intake (nPCR) during the first 6 months. This change remained stable (Table 1). Longer dialysis treatments allowed for reduced ultrafiltration rates, making it easier to achieve changes in the dry body weight compared to conventional treatment. The dry body weight showed an initial decrease after 3 months, from 68.3 ± 16.1 kg at baseline to 67.9 ± 15.7 kg and 68.2 ± 15.9 at 3 and 6 months, respectively, and gradually increased to 70.2 ± 16.1 kg at 24 months. The interdialytic weight gain increased from 2.3 ± 1 Kg at baseline to 2.7 ± 1.2 Kg at 24 months. The evolution of the nutritional biochemical parameters can be seen in Table 3.

### 3.4. Hematological Parameters and ESA Dose

The hemogram parameters and transferrin saturation remained stable, with all of them maintained within a normal range (Table 2). Ferritin increased after 3 months and remained stable from 354 ± 250 at baseline to 406 ± 255 ng/mL at 24 months. Inversely, iron and ESA doses decreased gradually, from 74.8 ± 52.1 at baseline to 56.2 ± 43.3 mg/week of iron at 24 months, and from 31.3 ± 20.5 at baseline to 16.7 ± 19.3 microg/week of ESA at 24 months. This reduction in ESA doses while maintaining hemoglobin levels resulted in a significant reduction in the erythropoietin resistivity index (ERI) from the sixth month onwards (Table 4).

### 3.5. Biochemical Parameters

Quarterly biochemical values are shown in Table 3. Bicarbonate levels were significantly higher in all patients compared with baseline values (from 23.8 ± 2.5 to 26.1 ± 2.4 mmol/L at 24 months). From the third month, blood–urea–nitrogen and creatinine values decreased and remained stable. However, there were no changes in sodium, potassium, magnesium, low-density lipoprotein cholesterol, high-density lipoprotein cholesterol, triglyceride, total protein, albumin, pre-albumin, β2-microglobulin, and glycated hemoglobin levels during the study period (Table 5).

### 3.6. Bone Profile

The pre-dialysis phosphorus levels decreased from a mean of 5.1 ± 1.6 to 3.6 ± 1.1 mg/dL over 24 months (Table 6). The addition of phosphorus supplements was carried out to avoid intradialytic hypophosphatemia, and it was required in 55% of the patients at 12 months and in 58% at 24 months. Conversely, phosphate binders were reduced from 3.28 ± 2.9 to 0.12 ± 0.6 tablets/day from the baseline to 24 months. No significant changes were observed in calcium, alkaline phosphatase, or intact parathyroid hormone, and the doses of paricalcitol and cinacalcet were unchanged (Table 7).

### 3.7. Blood Pressure Control

Excellent blood pressure control was observed. The mean systolic blood pressure, mean diastolic blood pressure, and mean blood pressure significantly decreased in a few months (Table 8). The pre-dialysis systolic blood pressure decreased from 139.8 ± 25.9 mm Hg at baseline to 125.4 ± 17.9 mm Hg at 24 months. Furthermore, antihypertensive treatment decreased from 1.07 ± 1.1 tablets/day to 0.5 ± 0.7 after 3 months and 0.37 ± 0.6 and 0.33 ± 0.5 tablets/day after 12 and 24 months, respectively.

### 3.8. Patient Survival

**Figure 2:**
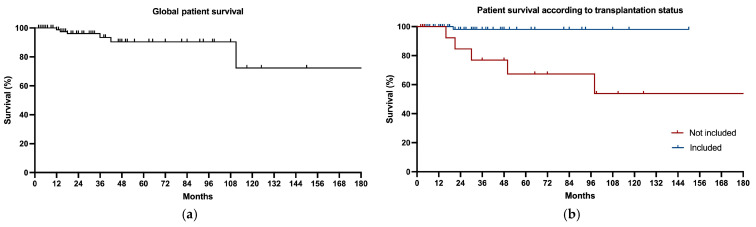
Patient survival. Global data (**a**) and differences according to transplantation status (**b**) are represented. Patients included (blue line) and not included (red line) on kidney transplantation waiting list.

The global patient survival is represented in Figure 2. It was 98% at 24 months and 94% at the end of the follow-up. The causes of the six deaths were oncological processes in three cases (liver carcinoma, gynecologic cancer, and colon cancer), one in the context of postoperative cardiac surgery, one from pneumonia, and one sudden death in a diabetic patient.

When this was divided up depending on inclusion or a lack of inclusion on the kidney transplantation waiting list, five deaths were seen in the non-included group (mortality rate of 31.25%) and only one patient died on the waiting list (mortality rate of 5.95%). So, the relative survival was 98.1% and 84.6% in included and non-included patients at 24 months, respectively, and 98.1% and 53.8% at the end of the follow-up (Figure 2).

### 3.9. Technique Survival

**Figure 3:**
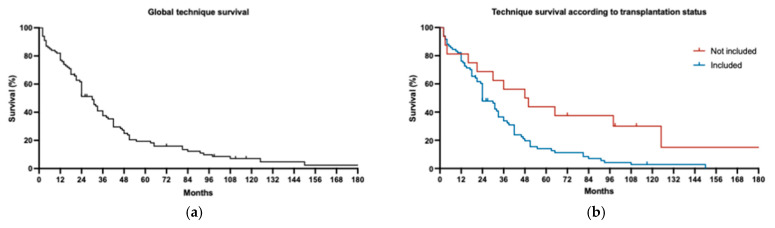
Technique survival. Global data (**a**) and differences according to transplantation status (**b**) are represented. Patients included on kidney transplantation waiting list (blue line) and not included (red line).

The median global technique survival is represented in Figure 3. It was 24 months with 60% of all the patients and it differed depending on the waiting list status, with the median technique survival of included and non-included patients being 24 months vs. 49 months, respectively. The technique survival rate was 48.7% and 81.3% at month 24, respectively, and 2.9% and 55.7% at month 120, respectively. The main cause of withdrawal was kidney transplantation in the group included on the waiting list (90.8%), while death was the main reason in the group not included on the waiting list (41.6%), and medical decisions were the second most frequent withdrawal cause in this group (33.3%).

## 4. Discussion

To our knowledge, this study represents one of the largest cohorts undergoing nocturnal, every-other-day OL-HDF, with one of the longest follow-up periods reported. This dialysis regimen was implemented in 2007 with the aim to enhance clinical outcomes by extending the treatment duration while also offering the convenience of overnight dialysis, which was expected to appeal to patients socially. Indeed, the results from these first 100 patients demonstrate high tolerance and acceptance, with effective social and occupational rehabilitation, as evidenced by the minimal voluntary withdrawals (three patients).

Regarding clinical outcomes, the dialysis dose, both in terms of diffusive and convective clearances, increased by nearly 60%. Additionally, this regimen was associated with a notably high patient survival rate. These findings align with our previous studies on smaller populations [18,19] and are consistent with other published reports highlighting the clinical benefits of (1) a longer dialysis duration, originally demonstrated by the Tassin group [20] and supported by subsequent studies [21,22,23,24,25,26], which have shown improved clinical outcomes, reduced mortality, and lower hospitalization rates; (2) more frequent dialysis schedules, as pioneered by the Lecce group [4] to avoid long weekend gaps; and (3) the enhanced removal of uremic toxins via convective therapies, recently confirmed by the CONVINCE trial [17], supporting the outcomes of the ESHOL study [16].

Over the 17-year period since the nocturnal dialysis program started, we observed significant clinical improvements in managing hyperphosphatemia, metabolic acidosis, anemia, blood pressure, and the nutritional status. Additionally, the need for phosphate binders, erythropoiesis-stimulating agents (ESAs), and antihypertensive medications decreased markedly.

Our findings regarding long-term dialysis outcomes are consistent with other studies addressing chronic kidney disease (CKD) complications. In terms of anemia management, we observed the stabilization of hematocrit levels alongside a 25% reduction in ESA doses and a 32% ESA discontinuation rate after 24 months. This mirrors findings from Poo et al. and Ok et al. [24,25] and may be attributed to the enhanced clearance of uremic toxins known to inhibit erythropoiesis [27].

Hyperphosphatemia, a well-established independent mortality risk factor due to its association with vascular calcification [28], was effectively managed in our cohort. We observed a sharp reduction in the use of phosphate binders from 76.67% to 3.3% after 24 months, accompanied by improved phosphate control. Interestingly, phosphorus supplementation in the dialysate was required in 59.3% of patients to prevent intradialytic hypophosphatemia. The residual renal phosphate clearance can be estimated using the volume of the urine output [29], but neither of those parameters was assessed in this study.

Hypertension, a prevalent condition among hemodialysis patients and a known mortality risk factor [30], was also effectively managed with extended dialysis. The need for antihypertensive medications was reduced by more than half (from 56.67% to 28.3% at 24 months), likely due to a reduced sympathetic tone [31], better clearance of pressor substances [31], and lower ultrafiltration rates, which help avoid intradialytic hypotension and fluid overload [32]. These results are consistent with previous publications [24,25]. Additionally, nocturnal dialysis has been associated with reduced left ventricular hypertrophy and improved cardiac function [33]. In our study, the dry weight decreased during the first 3 months (−0.9 kg), followed by stabilization, likely due to an improved nutritional status and lean mass gain. The blood pressure decreased significantly after 12 months. Beyond clinical outcomes, nocturnal hemodialysis offers the flexibility of overnight treatment, enabling patients to engage in daytime activities, including employment, thereby improving the overall quality of life. Previous studies have shown that switching to nocturnal schedules improves the mental health-related quality of life [34], a finding supported by the high patient acceptance and low dropout rate in our cohort. Additionally, the lower technical survival in transplant-listed patients may reflect the higher rate of transplantation in this group, which remains the most common cause of dialysis cessation in transplant-eligible patients. Conversely, patients not on the transplant waiting list tend to have more comorbidities, contributing to higher mortality rates.

From an economic perspective, implementing a nocturnal dialysis program entails additional costs associated with extended shift durations (from 7 to 10 h) and the payment of a night shift premium to staff. However, these additional costs can be mitigated due to the clinical stability of the patients, which allowed for maintaining a nurse-to-patient ratio of 1:5 instead of 1:4. Furthermore, potential emergencies were managed by the on-call physician, who was already present in the hospital as part of their routine duties.

This study has some limitations. Patients who are candidates for nocturnal schedules tend to be younger, more active, and of working age, introducing a selection bias. Moreover, this is a descriptive study without a control group of patients on conventional dialysis schedules for comparison. Additionally, Spain has one of the highest kidney transplantation rates globally, which may limit the generalizability of our findings to countries with different transplantation systems or criteria. However, given the shorter waiting times for transplantation in Spain, our results may actually underscore the potential for even greater benefits in countries with longer wait times for kidney transplants.

## 5. Conclusions

In conclusion, this large, single-center cohort of patients treated with nocturnal, every-other-day OL-HDF with high convective volumes demonstrated improved clinical outcomes related to key risk factors for cardiovascular comorbidity and mortality, including hyperphosphatemia, hypertension, and anemia. Furthermore, this treatment regimen exhibited excellent technical survival and was well accepted by patients, with low withdrawal and mortality rates. Our team remains committed to the demonstrated benefits of long, frequent nocturnal OL-HDF and aims to advocate for its broader adoption in clinical practice.

## Figures and Tables

**Table 1:** Dialysis parameters and convective volume evolution.

Variable	Baseline	3 m	6 m	9 m	12 m	15 m	18 m	21 m	24 m
Time (min)	279 ± 28	479 ± 8 ^a^	478 ± 11 ^a^	478 ± 11 ^a^	478 ± 11 ^a^	478 ± 11 ^a^	478 ± 11 ^a^	478 ± 11 ^a^	478 ± 11 ^a^
Qb (mL/min)	422 ± 34	421 ± 34	416 ± 31 ^f^	415 ± 32 ^f^	415 ± 32 ^f^	416 ± 33	416 ± 33	416 ± 33	415 ± 34 ^f^
Qd (mL/min)	516 ± 173	348 ± 112 ^a^	356 ± 109 ^a^	255 ± 109 ^a^	361 ± 106 ^a^	349 ± 106.9 ^a^	354 ± 106 ^a^	357 ± 104 ^a^	359 ± 104 ^a^
Convective volume (L)	27.1 ± 4.6	42.7 ± 10.3 ^a^	44.1 ± 9.7 ^a^	43.4 ± 10.5 ^a^	43.6 ± 10.8 ^a^	46.9 ± 6.6 ^a,e^	47.2 ± 6.2 ^a,e^	47.4 ± 7.2 ^a,e^	48.1 ± 6.4 ^a,e^
Initial weight (Kg)	70.5 ± 16.3	70.7 ± 16.2	71.0 ± 16.2	71.7 ± 16.3 ^c,e^	72.2 ± 16.4 ^b^	72.7 ± 16.3 ^a^	72.6 ± 16.4 ^b,f^	72.9 ± 16.7 ^b,f^	72.9 ± 16.4 ^b,f^
Final weight (Kg)	68.2 ± 16.1	67.9 ± 15.7	68.2 ± 15.9	68.7 ± 16 ^f^	69.3 ± 15.9 ^e^	70.0 ± 15.9 ^b,d^	70.0 ± 16.2 ^b,d^	70.0 ± 16.3 ^b,d^	70.1 ± 16.1 ^b,d^
Weight gain (Kg)	2.3 ± 1.0	2.8 ± 1.1 ^b^	2.8 ± 0.9 ^b^	2.9 ± 1.0 ^a^	2.9 ± 1.1 ^a^	2.7 ± 1.0 ^b^	2.6 ± 1.1	2.8 ± 1.0 ^b^	2.7 ± 1.2 ^c^

Variables are expressed as mean ± standard deviation. L: liters; m: months; Qb: blood flow; Qd: dialysis liquid flow. ^a^: *p* < 0.001; ^b^: *p* < 0.01; ^c^: *p* < 0.05 with respect to baseline value (analysis of variance, repeated measures); ^d^: *p* < 0.001; ^e^: *p* < 0.01; ^f^: *p* < 0.05 with respect to month 3.

**Table 2:** Dialysis dose.

Variable	Baseline	3 m	6 m	9 m	12 m	15 m	18 m	21 m	24 m
Kt (L)	67.6 ± 12.0	103.8 ±14.2 ^a^	103.5 ±10.7 ^a^	103.1 ± 11.8 ^a^	102.6 ± 12.2 ^a^	104.1 ± 10.1 ^a^	104.4 ± 10.5 ^a^	105.1 ± 12.9 ^a^	105.4 ± 11.7 ^a^
TAC (mg/dL)	30.8 ± 8.0	24.1 ± 6.5 ^a^	25.0 ± 6.7 ^a^	25.2 ± 7.2 ^a^	24.3 ± 7.4 ^a^	24.6 ± 6.3 ^a^	23.1 ± 6.7 ^a^	23.9 ± 6.0 ^a^	24.4 ± 6.9 ^a^
URR (%)	80.1 ± 5.6	88.4 ± 5.5 ^a^	88.7 ± 4.9 ^a^	88.7 ± 4.3 ^a^	89.1 ± 3.8 ^a^	88.4 ± 4.3 ^a^	88.4 ± 4.2 ^a^	89.2 ± 3.6 ^a^	88.5 ± 4.3 ^a^
spKt/V	2.01 ± 0.39	3.54 ± 1.00 ^a^	3.65 ± 1.08 ^a^	3.64 ± 1.07 ^a^	3.72 ± 1.21 ^a^	3.50 ± 1.02 ^a^	3.50 ± 0.98 ^a^	3.64 ± 0.92 ^a^	3.47 ± 1.00 ^a^
eKt/V	1.72 ± 0.33	3.09 ± 0.87 ^a^	3.20 ± 0.94 ^a^	3.18 ± 0.94 ^a^	3.25 ± 1.06 ^a^	3.06 ± 0.89 ^a^	3.06 ± 0.85 ^a^	3.19 ± 0.81 ^a^	3.04 ± 0.87 ^a^
stdKt/V	2.54 ± 0.20	3.69 ± 0.28 ^a^	3.72 ± 0.20 ^a^	3.72 ± 0.22 ^a^	3.72 ± 0.23 ^a^	3.69 ± 0.22 ^a^	3.69 ± 0.23 ^a^	3.74 ± 0.19 ^a^	3.68 ± 0.25 ^a^
nPCR	1.19 ± 0.33	1.28 ± 0.39	1.39 ± 0.55 ^b^	1.33 ± 0.53 ^c^	1.31 ± 0.48	1.31 ± 0.41 ^c^	1.24 ± 0.41 ^c^	1.28 ± 0.37	1.29 ± 0.41

Variables are expressed as mean ± standard deviation. TAC: time-averaged concentration; URR: urea reduction ratio; spKt/V, single-pool Kt/V; eKt/V, equilibrated Kt/V; stdKt/V, standard Kt/V; nPCR: normalized protein catabolic rate. ^a^: *p* < 0.001; ^b^: *p* < 0.01; ^c^: *p* < 0.05 with respect to baseline value (analysis of variance, repeated measures).

**Table 3:** Evolution of nutritional biochemical parameters.

Variable	Baseline	3 m	6 m	9 m	12 m	15 m	18 m	21 m	24 m
Creatinine (mg/dL)	7.49 ± 1.77	6.27 ± 1.12 ^a^	6.40 ± 1.35 ^a^	6.45 ± 1.25 ^a^	6.51 ± 1.12 ^b^	6.64 ± 1.16 ^b^	6.69 ± 1.29 ^c^	6.62 ± 1.30 ^b^	6.56 ± 1.29 ^b^
Total cholesterol (mg/dL)	168.7 ± 37.6	171.9 ± 32.3	176.4 ± 41.0	176.7 ± 46.3	173.4 ± 41.9	173.6 ± 38.8	170.9 ± 40.5	169.7 ± 40.7	166.8 ± 37.4
LDL (mg/dL)	96.7 ± 32.4	92.5 ± 24.2	95.3 ± 32.5	96.3 ± 31.4	90.8 ± 30.3	94.1 ± 28.5	95.7 ± 31.5	93.1 ± 29.4	88.5 ± 25.1
HDL (mg/dL)	44.4 ± 18.6	45.6 ± 17.6	47.0 ± 31.0	46.6 ± 19.4	48.7 ± 42.9	48.0 ± 42.6	47.6 ± 35.7	48.9 ± 39.4	45.0 ± 23.5
Triglycerides (mg/dL)	152 ± 103	205 ± 156	183 ± 111	190 ± 108	205 ± 116 ^b^	197 ± 118 ^c^	171 ± 82	184 ± 104	189 ± 103
Total proteins (g/L)	6.49 ± 0.54	6.46 ± 0.51	6.52 ± 0.51	6.46 ± 0.49	6.54 ± 0.56	6.48 ± 0.56	6.54 ± 0.51	6.47 ± 0.56	6.51 ± 0.47
Albumin (g/L)	3.91 ± 0.38	3.81 ± 0.35 ^c^	3.85 ± 0.32	3.82 ± 0.27	3.90 ± 0.32 ^f^	3.86 ± 0.33	3.87 ± 0.35	3.84 ± 0.37	3.85 ± 0.35
Pre-albumin (g/L)	0.3 ± 0.06	0.28 ± 0.07 ^a^	0.31 ± 0.08 ^e^	0.31 ± 0.06 ^e^	0.31 ± 0.06 ^d^	0.3 ± 0.07	0.31 ± 0.07	0.29 ± 0.07	0.3 ± 0.07

LDL: low-density lipoprotein; HDL: high-density lipoprotein. ^a^: *p* < 0.001; ^b^: *p* < 0.01; ^c^: *p* < 0.05 with respect to baseline value (analysis of variance, repeated measures); ^d^: *p* < 0.001; ^e^: *p* < 0.01; ^f^: *p* < 0.05 with respect to month 3.

**Table 4:** Evolution of hematological parameters.

Variable	Baseline	3 m	6 m	9 m	12 m	15 m	18 m	21 m	24 m
Hemoglobin (g/dL)	11.45 ± 1.67	11.63 ± 1.50	12.01 ± 1.44 ^c^	11.61 ± 1.64	11.88 ± 1.31	11.81 ± 1.26	11.64 ± 1.24	11.41 ± 1.20	11.53 ± 1.16
Hematocrit (%)	35.7 ± 5.1	35.6 ± 4.5	36.6 ± 4.3	35.6 ± 4.8	36.1 ± 4.1	36.3 ± 3.7	35.9 ± 3.8	34.9 ± 4.1	35.5 ± 3.9
Leucocytes (×10^9^)	6.78 ± 2.18	7.11 ± 1.94	7.18 ± 1.79	7.28 ± 2.28 c	7.05 ± 2.06	7.16 ± 2.09	7.05 ± 2.03	6.92 ± 1.83	6.80 ± 1.81
Platelets (×10^9^)	219.4 ± 70.2	215.2 ± 62.1	212.3 ± 66.8	213.8 ± 72.8	209.5 ± 53.1	209.4 ± 66.3	212.3 ± 61.7	213.9 ± 65.3	220.3 ± 71.01
TS (%)	22.9 ± 9	21.2 ± 7.1	22.7 ± 7.2	21.6 ± 8.1	20.6 ± 7	22.3 ± 7.5	21.0 ± 7.4	21.2 ± 8.1	21.9 ± 8.4
Ferritin (ng/mL)	354 ± 250	444 ± 297 ^c^	466 ± 274 ^b^	486 ± 297 ^b^	476 ± 274 ^c^	466 ± 332	442 ± 301	415 ± 289	405 ± 254
Transferrin (g/L)	1.87 ± 0.35	1.82 ± 0.33	1.84 ± 0.32	1.89 ± 0.35	1.89 ± 0.37	2.09 ± 1.87	2.49 ± 2.98	2.43 ± 2.70	2.39 ± 2.53
Iron dose (mg/week)	74 ± 52	99 ± 76 ^b^	77 ± 68 ^f^	68 ± 60 ^e^	68 ± 70 ^e^	61 ± 66 ^e^	55 ± 49 ^c,d^	57 ± 38 ^c,d^	56 ± 43 ^c,d^
ESA dose (IU/week)	6266 ± 4100	5716 ± 4240	4634 ± 4060 ^b,f^	4384 ± 4360 ^b,e^	4384 ± 4440 ^b,f^	3750 ± 4220 ^c,d^	3566 ± 4000 ^c,d^	3450 ± 3840 ^c,d^	3350 ± 3860 ^c,d^
ERI (IU/week/Kg/g Hb)	8.91 ± 6.99	8.07 ± 7.08	6.32 ± 6.45 ^a,e^	6.54 ± 7.91 ^b,f^	6.03 ± 6.61 ^a,e^	5.17 ± 6.25 ^a,d^	5.02 ± 5.98 ^a,e^	4.87 ± 5.76 ^a,d^	4.64 ± 5.80 ^a,d^

TS, transferrin saturation; ESA: erythropoiesis-stimulating agents; ERI: erythropoietin resistivity index (ESA doses/hemoglobin); IU: international unit. ^a^: *p* < 0.001; ^b^: *p* < 0.01; ^c^: *p* < 0.05 with respect to baseline value (analysis of variance, repeated measures); ^d^: *p* < 0.001; ^e^: *p* < 0.01; ^f^: *p* < 0.05 with respect to month 3.

**Table 5:** Evolution of other biochemical parameters.

Variable	Baseline	3 m	6 m	9 m	12 m	15 m	18 m	21 m	24 m
C-reactive protein (mg/dL)	1.1 ± 2.1	1.0 ± 1.1	0.8 ± 1.4	1.1 ± 2.2	0.8 ± 1.0	1.0 ± 1.8	0.7 ± 0.9	0.8 ± 1.0	0.9 ± 1.6
Blood–urea–nitrogen (mg/dL)	50.85 ± 13.5	43.76 ± 12.2	45.24 ± 12.1	45.31 ± 13.5	43.81 ± 12.6	45.18 ± 14.1	42.42 ± 12.5	43.00 ± 11.3	43.95 ± 13.2
Uric acid (mg/dL)	6.63 ± 1.31	6.37 ± 1.27	6.33 ± 1.09	6.47 ± 1.18	6.41 ± 0.97	6.33 ± 0.88	6.33 ± 1.08	6.26 ± 1.13	6.14 ± 0.94
Sodium (mEq/L)	140.1 ± 2.7	139.8 ± 3.1	139.4 ± 2.9	139.4 ± 3.3	139.9 ± 3.0	140.1 ± 2.8	140.5 ± 2.7	140.5 ± 2.7	140.2 ± 2.8
Potassium pre-dialysis (mEq/L)	4.74 ± 0.79	4.31 ± 0.79 ^a^	4.55 ± 0.80	4.46 ± 0.73	4.56 ± 0.71	4.38 ± 0.60	4.36 ± 0.60 ^c^	4.46 ± 0.64	4.41 ± 0.65
Potassium post-dialysis	3.14 ± 0.35	3.12 ± 0.51	3.15 ± 0.43	3.09 ± 0.44	3.08 ± 0.40	3.08 ± 0.36	3.11 ± 0.38	3.11 ± 0.45	3.11 ± 0.42
Glycated hemoglobin (%)	5.3 ± 1.5	5.3 ± 1.5	5.3 ± 1.2	5.3 ± 1.3	5.2 ± 1.0	5.3 ± 1.1	5.3 ± 1.2	5.4 ± 1.4	5.4 ± 1.2
Serum bicarbonate (mmol/L)	23.8 ± 2.5	25.3 ± 2.8 ^a^	25.8 ± 3.2 ^a^	25.4 ± 3.0 ^a^	25.6 ± 3.0 ^a^	26.1 ± 2.9 ^a^	26.2 ± 2.7 ^a^	26.4 ± 3.1 ^a,d^	26.1 ± 2.4 ^a,e^
ß_2_-microglobulin (mg/L)	27.2 ± 10.3	24.5 ± 7.1 ^b^	24.9 ± 8.7 ^c^	25.8 ± 9.8	25.8 ± 8.6 ^e^	26.2 ± 8.3 ^e^	24.5 ± 7.5 ^b^	25.6 ± 7.8	25.2 ± 7.3

^a^: *p* < 0.001; ^b^: *p* < 0.01; ^c^: *p* < 0.05 with respect to baseline value (analysis of variance, repeated measures); ^d^: *p* < 0.01; ^e^: *p* < 0.05 with respect to month 3.

**Table 6:** Evolution of phosphor–calcium parameters.

Variable	Baseline	3 m	6 m	9 m	12 m	15 m	18 m	21 m	24 m
Calcium (mg/dL)	8.8 ± 0.6	8.9 ± 0.7	8.9 ± 0.4	8.9 ± 0.5	8.9 ± 0.5	8.8 ± 0.7	8.9 ± 0.6	8.8 ± 0.6	8.8 ± 0.8
Pre-dialysis phosphorus (mg/dL)	5.1 ± 1.6	3.7 ± 1.2 ^a^	3.9 ± 1.3 ^a^	3.6 ± 1.2 ^a^	3.6 ± 1.1 ^a^	3.6 ± 1.1 ^a^	4.2 ± 1.0	4.1 ± 1.2 ^b^	3.6 ± 1.1 ^a^
Post-dialysis phosphorus (mg/dL)	2.1 ± 0.4	1.7 ± 0.5 ^a^	1.7 ± 0.4 ^a^	1.7 ± 0.4 ^a^	1.7 ± 0.3 ^a^	1.8 ± 1.1	2.0 ± 2.6	1.9 ± 2.2	2.0 ± 2.5
Magnesium (mg/dL)	2.27 ± 0.29	2.24 ± 0.23	2.29 ± 0.32	2.23 ± 0.22	2.27 ± 0.24	2.25 ± 0.25	2.24 ± 0.22	2.19 ± 0.18	2.19 ± 0.22
iPTH (pg/mL)	361 ± 295	285 ± 306	275 ± 336	309 ± 366	341 ± 394	324 ± 213	300 ± 169	310 ± 214	295 ± 198
Alkaline phosphatase (U/L)	199 ± 215	224 ± 227	214 ± 238	212 ± 213	214 ± 197	220 ± 213	215 ± 193	192 ± 156	191 ± 151

iPTH: intact parathormone. ^a^: *p* < 0.001; ^b^: <0.05 with respect to baseline value (analysis of variance, repeated measures).

**Table 7:** Evolution of the use of phosphate binders, vitamin D analogs, and calcimimetic doses. Dialysate calcium prescription and dialysate phosphate supplements needed.

Variable	Baseline	3 m	6 m	9 m	12 m	15 m	18 m	21 m	24 m
Phosphate binders (*n*)	3.2 ± 2.9	0.1 ± 0.4 ^a^	0.01 ± 0.4 ^a^	0.2 ± 0.7 ^a^	0.2 ± 0.7 ^a^	0.2 ± 0.7 ^a^	0.2 ± 0.7 ^a^	0.1 ± 0.6 ^a^	0.1 ± 0.6 ^a^
Phosphorus binders needed (%)	76.67	3.33	3.33	6.66	6.66	6.66	6.66	5.00	3.33
Calcimimetic dose (mg/week)	9.3 ± 19.9	7.8 ± 17.5	8.7 ± 18.9	10.0 ± 20.9	10.5 ± 22.4	12.5 ± 24.0 ^b^	13.3 ± 25.3 ^b^	11.3 ± 21.8	10.3 ± 20.9
Vitamin D analogs (*n*)	2.0 ± 2.7	2.1 ± 3.0	2.1 ± 3.1	1.6 ± 2.2	1.7 ± 2.5	1.5 ± 2.1	1.9 ± 2.5	1.8 ± 2.5	1.8 ± 2.4
Calcium in dialysate, 1.5 mmol/L/1.25 mmol/L (%)	97/3	95/5	98/2	95/5	88/12	86/14	90/10	90/10	88/12
Phosphate dialysate supplements (%)	0	42	55	55	55	57	55	58	58

^a^: *p* < 0.001 with respect to baseline value (analysis of variance, repeated measures); ^b^: *p* < 0.05 with respect to month 3.

**Table 8:** Evolution of blood pressure parameters.

Variable	Baseline	3 m	6 m	9 m	12 m	15 m	18 m	21 m	24 m
Systolic BP (mmHg)	140 ± 26	133 ± 20 ^c^	131 ± 23 ^c^	130 ± 22 ^b^	124 ± 21 ^a,e^	124 ± 21 ^a,d^	126 ± 22 ^b,e^	129 ± 22 ^b^	126 ± 18 ^a,e^
Diastolic BP (mmHg)	75 ± 16	71 ± 13 ^c^	70 ± 14 ^c^	69 ± 13 ^c^	68 ± 13 ^c^	68 ± 14 ^b^	68 ± 15 ^c^	68 ± 13 ^b^	66 ± 13 ^a,d^
Antihypertensives (*n*)	1.0 ± 1.1	0.5 ± 0.7 ^a^	0.5 ± 0.7 ^a^	0.4 ± 0.6 ^a^	0.4 ± 0.6 ^a,f^	0.3 ± 0.5 ^a,f^	0.3 ± 0.5 ^a,f^	0.3 ± 0.5 ^a,f^	0.3 ± 0.5 ^a,f^
Antihypertensive use (%)	56.7	35.0	35.0	30.0	28.3	28.3	28.3	28.3	28.3

Variables are expressed as mean ± standard deviation. BP: blood pressure; mmHg: millimeters of mercury. Kg: kilogram. ^a^: *p* < 0.001; ^b^: *p* < 0.01; ^c^: *p* < 0.05 with respect to baseline value (analysis of variance, repeated measures); ^d^: *p* < 0.001; ^e^: *p* < 0.01; ^f^: *p* < 0.05 with respect to month 3.

## Data Availability

The data that support the findings of this study are available from the corresponding author upon reasonable request.
